# Cardiac papillary fibroelastoma involving the mitral valve: A case report

**DOI:** 10.1097/MD.0000000000041296

**Published:** 2025-01-17

**Authors:** Yuqiong An, Fang Nie

**Affiliations:** a Ultrasound Medical Center, Second Hospital of Lanzhou University, Lanzhou, China.

**Keywords:** case report, echocardiography, surgery, valve tumor

## Abstract

**Rationale:**

Cardiac papillary fibroelastoma (CPF) is a rare cardiac tumor that can lead to severe and potentially fatal complications such as stroke, myocardial infarction, and sudden cardiac death. The rarity of CPF makes it challenging for clinicians to diagnose and treat, highlighting the importance of timely and accurate diagnosis to prevent catastrophic outcomes. This case report aims to contribute to the clinical understanding of CPF involving the mitral valve (MV), providing insights into diagnosis and treatment strategies.

**Patient concerns:**

We present a case of CPF attached to the MV in a 69-year-old male patient with a 3-year history of syncope.

**Diagnoses:**

Echocardiography and computed tomography (CT) imaging confirmed a floating, round-shaped mass on the anterior leaflet MV, with pathological analysis supporting the diagnosis of CPF.

**Interventions:**

The patient underwent the tumor resection and valve repair operation.

**Outcomes:**

Postoperative recovery was favorable, with no evidence of recurrence or valve dysfunction at 1-year follow-up.

**Lessons:**

Echocardiography is a valuable diagnostic tool for identifying CPF and guide treatment decisions. This case emphasizes the importance of recognizing this rare valve tumor for timely clinical intervention.

## 1. Introduction

Cardiac papillary fibroelastoma (CPF), a relatively rare primary benign tumor of the heart, is the most prevalent type of cardiac valve tumor, accounting for approximately 90% of all valve tumors.^[1]^ It can develop on any endocardial surface but predominantly affects cardiac valves (88%),^[2]^ with the aortic valve being the most commonly involved, followed by the mitral valve (MV), tricuspid valve and pulmonary valve.^[3]^ The first case of CPF was reported by Cheitlin et al in 1975.^[4]^ Due to its low incidence and limited examination methods, there have been only a few related reports since then. However, CPF poses a life-threatening risk as it can lead to fatal embolic complications such as cerebral stroke, myocardial infarction, and sudden cardiac death.^[5]^ Therefore, early detection of CPF and timely implementation of appropriate surgical intervention are crucial. In our case, echocardiography has proven to be an excellent technique for confirming the diagnosis of CPF and providing valuable insights for surgical planning.

## 2. Case report

**Figure 1.**
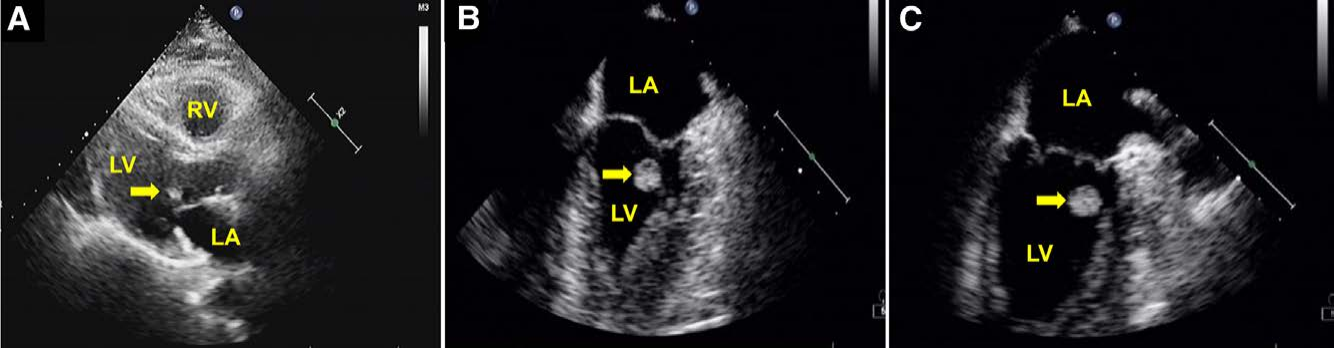
(A) Two-dimensional transthoracic echocardiography showing a floating round-shaped hyperechoic mass on the anterior mitral valve (yellow arrow). (B, C) Transesophageal echocardiography showing a regular, round tumor (yellow arrow) that appeared to be attached to the anterior mitral valve leaflet by a small pedicle. LA = left atrium, LV = left ventricle, RA = right atrium.

**Figure 2.**
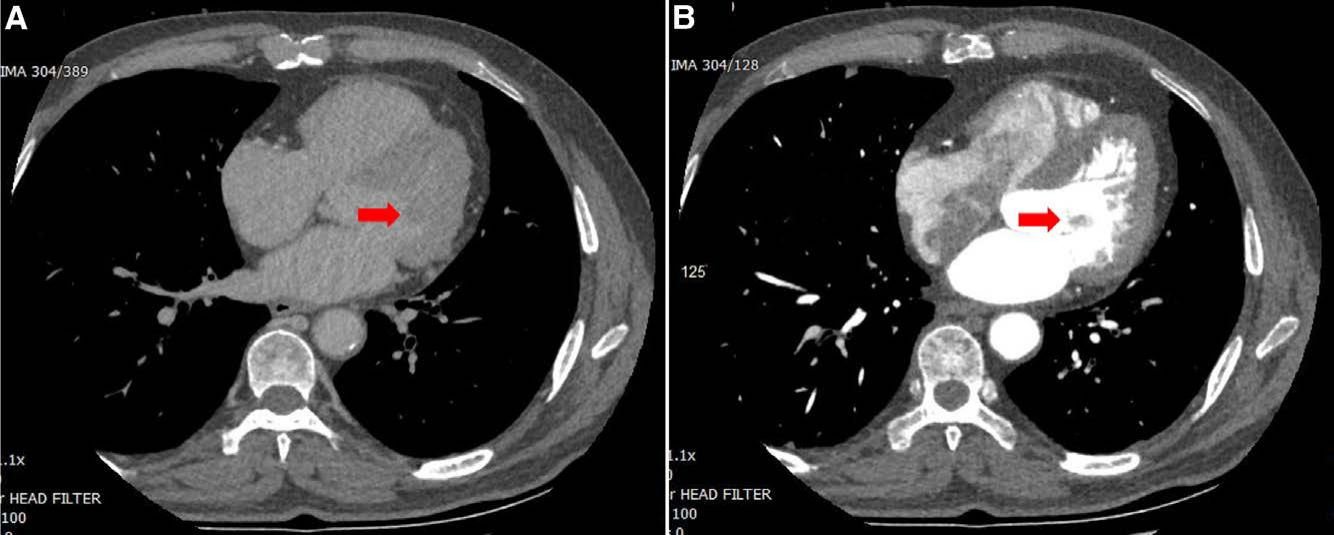
Noncontrast (A) and contrast-enhanced CT (B) imaging of the heart revealed an oval hypodense lesion (red arrow), which appeared to be attached to the anterior mitral valve leaflet by a small pedicle.

The patient, a 69-year-old man with a history of syncope over the past 3 years, presented to our hospital. Upon admission, the patient was hemodynamically stable, showed normal markers of myocardial necrosis and no electrocardiogram abnormalities. Auscultation of the heart revealed no murmurs. Subsequently, an integrated imaging assessment combining echocardiography and cardiac computed tomography (CT) was performed. Transthoracic echocardiography (TTE) revealed a floating round-shaped hyperechoic mass measuring 11 × 10 mm on the anterior MV, as shown in Figure 1A. Transesophageal echocardiographic (TEE) imaging depicted a regular, round tumor that appeared to be attached to the anterior leaflet MV by a small pedicle (Fig. 1B, C). Noncontrast (Fig. 2A) and contrast-enhanced CT (Fig. 2B) imaging of the heart revealed an oval hypodense lesion, which appeared to be attached to the anterior mitral valve leaflet by a small pedicle. After discussion with the patient’s family members, the surgeon decided to proceed with resection of the mass.

**Figure 3.**
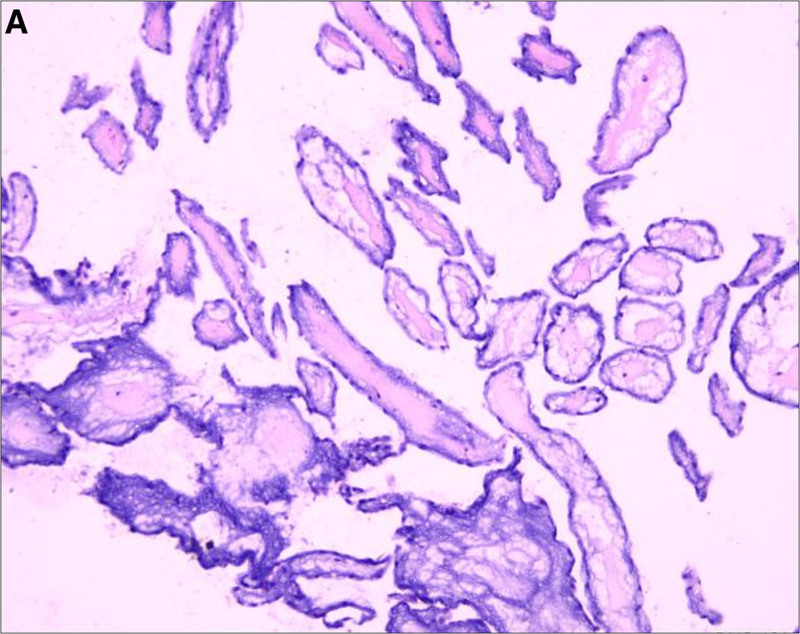
Histological examination showed the branching avascular papilla, composed of the hyalinized collagen matrix, which were covered by the endothelium.

The surgery was performed under general endotracheal anesthesia via total median sternotomy. During the procedure, a tumor was identified on the MV, specifically attached to the middle part of the anterior cusp by a pedicle. Subsequently, tumor resection and valve repair were conducted. Histopathological examination of the specimen revealed mesenchymal tissue exhibiting characteristics consistent with CPF, as demonstrated in Figure 3. The patient was discharged in good condition 8 days postsurgery. Follow-up transthoracic echocardiography 1 year after surgery showed no abnormal echoes on the MV, with no signs of mitral stenosis or regurgitation, indicating successful tumor resection and valve repair. The written informed consent was obtained from the patient’s next of kin for the use of the clinical data and for the publication of this case report. Considering the retrospective nature of the study and the use of anonymized data, formal ethics approval was waived. All methods were performed in accordance with the Declaration of Helsinki.

## 3. Discussion and conclusions

Primary cardiac tumors (PCT) are rare findings in autopsies, reported in only 0.02% of cases. Cardiac papillary fibroelastomas (CPF) rank as the third most common PCT, accounting for approximately 8% of all PCT cases. Nevertheless, a large registry study recently suggests that the incidence of CPF may be higher than any other type of PCT. In this article, we present a case of an echocardiography-detected CPF on the MV, following tumor resection and valve repair surgery, the patient has shown a favorable prognosis.

### 3.1. Pathophysiology

CPF typically presents as solitary tumors, usually measuring <20 mm in size. The tumor appears milky white and has a soft texture, with a villous or cauliflower-like surface. When placed in water, it exhibits a distinct sea anemone-like appearance. Histologically, CPFs are avascular and consist of a single layer of endocardial cells covering the papillary surface. The matrix is composed of irregular elastic fibers, proteoglycans, and spindle cells resembling smooth muscle cells or fibroblasts. The presence of this layer of elastic fibers is a characteristic feature of this tumor type. The exact histogenesis of CPF remains unclear; however, hypotheses regarding its etiology include neoplasm formation, harmatomas development, organized thrombi formation, and an unusual endocardial response to infection or iatrogenic causes.^[6]^ Among these theories, the microthrombus theory is widely accepted and suggests that small thrombi coalesce on the coapting margins of valves at sites with minor endothelial damage.

### 3.2. Clinical presentation

The clinical presentation of CPF depends on various factors such as tumor location, size, growth rate, and propensity for embolization. Symptoms can range from none to severe thromboembolic complications including transient ischemic attack, strokes, and myocardial infarction. Other potential complications include pulmonary embolism, congestive heart failure, syncope, and sudden death. Due to over 95% occurrence on the left side of the heart, the most common manifestation is systemic embolism. CPF has been reported to cause embolization to the coronary arteries, lungs, brain, kidneys, retain a spinal cord, mesentery vessels, and lower limbs. In cases where right-sided CPF occurs, pulmonary emboli are more frequent which may lead to pulmonary hypertension and death.

### 3.3. Diagnosis

CPF can manifest at any age, although it is most commonly observed in middle-aged and older adults. Pathological confirmation is essential for a definitive diagnosis of CPF. Prior to the introduction of echocardiography, the detection rate of CPF was extremely low. Currently, echocardiography plays a crucial role in diagnosing preoperative CPF due to its distinctive features, including predominantly affecting cardiac valves, with the aortic valve being the most frequently involved followed by the mitral, tricuspid, and pulmonary valves; presenting as round and echo-dense tumors that are often pedunculated with high-frequency oscillations during the cardiac cycle; rarely impacting valve function or causing stenosis or regurgitation. While transthoracic echocardiography (TTE) is effective in detecting large tumor volumes, transesophageal echocardiography (TEE) can be employed as an adjunctive examination when dealing with smaller tumor volumes. The role of computed tomography (CT), which has limited soft tissue differentiation capabilities and involves exposure to ionizing radiation, remains uncertain regarding CPF diagnosis. In this case study, we successfully visualized the shape and boundaries of a tumor on the anterior leaflet of the mitral valve through combined TTE and TEE imaging.

### 3.4. Differential diagnosis

The differential diagnosis for CPF includes cardiac myxomas, lambl’s excrescence (LED), infective endocarditis (IE), thrombus, and so on. Cardiac myxomas: The cardiac myxoma is the most common primary tumor of the heart. When the CPF attached to endocardial surface should be distinguished with cardiac myxoma. However, the myxomas rarely occur on valves, and its volume is usually large and its shape is often irregularity. LED: LED tends to be multiple tumors, usually have a cord-like appearance. LEDs are often located on the atrial surface of the atrioventricular valve closure. Semilunar valve can be located anywhere, but it is rare to be located on the arterial surface of the valve. IE: the bacterial vegetations are often band-like or lumpy, with different shapes, and often occur in the site of high-speed blood flow impact. IE also associated with stigmata of bacterial infection, including leukocytosis, positive blood cultures, and other systemic signs of infection. It is not difficult to diagnose combined with medical history. Cardiac thrombus: thrombus often occurs where blood flow is slow. It can be comprehensively diagnoses by combining concomitant lesions and hemodynamic status.

### 3.5. Treatment

Surgical excision is the definitive treatment for symptomatic CPF.^[7]^ Once diagnosed, prompt surgical resection should be performed to mitigate the risk of syncope or sudden death resulting from tumor embolism. In cases where the tumor is small and located near the free edge of the valve, a combination of tumor resection and valve repair may be considered. However, if the tumor is large or situated close to the base of the valve, valve replacement is often necessary. Furthermore, due to its pedunculated nature, CPF can be easily removed during surgery with minimal recurrence rates and favorable postoperative outcomes.

### 3.6. Study Limitations

This case report is limited by its single-case design, which restricts the generalizability of the findings. The rarity of CPF also limits the ability to draw broad conclusions from a single patient experience. Future studies with larger sample sizes are needed to validate the diagnostic and surgical approaches presented here.

In Conclusion, echocardiography’s accessibility, repeatability, and lack of radiation exposure make it an invaluable tool in diagnosing CPF and guiding treatment decisions.

## Acknowledgments

We would like to express our gratitude to the patient for permission to use these clinical data and for the publication of this research.

## Author contributions

**Supervision:** Fang Nie.

**Writing – original draft:** Yuqiong An.
